# A Manual of Bandaging

**Published:** 1885-01

**Authors:** 


A MANUAL OF BANDAGING, adapted for self-instruction. BY C. HENRI LEONARD, A. M., M.D., Professor of the Medical and Surgical Diseases of Women, and Clinical Gynæcology, Michigan College of Medicine; member of the American Medical Association; of the Michigan State Medical Society; of the Wayne County Medical Society; and honorary member of the Canada Medical Association. With one hundred and thirty-nine engravings, second edition, revised and enlarged. Published by The Illustrated Medical Journal Co., Detroit.

While this description, with drawings of many bandages which are requisite in dressing different injuries, meets a very sensible want, it is rather surprising that nothing appears in respect to the Esmarch bandage and some others which are recognized of great value in surgical proceedings.

While the old complicated bandage of Velpeau, for fractured clavicle is retained, it is a subject of congratulation that another time-honored gearing known as Dessault's bandage, has ceased to have a place in the apparatus for fractured clavicle.

The adhesive plaster bandage of Sayre, if properly applied, ought to meet the indications well, but the horizontal waistb-and should pass around the arm near the elbow instead of being at its middle. In the absence of such material as adhesive plaster two ordinary pocket handkerchiefs may be so applied around the elbow as that one passing over the neck and the other circling the body with a pad in the armpit, shall accomplish the lifting of the shoulder on the injured side, and by drawing in the elbow carry the head of the humerus, with its attachments, outward, which are the prime objects to be fulfilled in obviating the deformity in dropping of the shoulder from fracture of the clavicle. Simplicity, with efficiency, are the prime elements for consideration in a choice of bandages, and all such complicated turns of the roller as are represented in the three different crosses for the mamma may be substituted by simpler appliances which meet the same indications.

If the author had given us some of the plain figures from Sedillot's little work, issued thirty years ago, in place of such elaborate applications as are depicted, it should not be regarded as detracting from the value of the work, though progressing backwards.

In a work intended for the instruction of those removed from the great centres of surgical appliances, simplicity is essential for the proper execution of bandaging; and while the refinements which are so conspicuous in this manual are doubtless for the most part effective, when properly applied, it requires a preparatory training which few general practitioners have enjoyed for turning them to account. Yet the work is well gotten up and reflects credit on the author.

